# Stock price movement prediction based on Stocktwits investor sentiment using FinBERT and ensemble SVM

**DOI:** 10.7717/peerj-cs.1403

**Published:** 2023-06-07

**Authors:** Jin-Xian Liu, Jenq-Shiou Leu, Stefan Holst

**Affiliations:** 1 Department of Electronic and Computer Engineering, National Taiwan University of Science and Technology, Taipei, Taiwan; 2 Department of Computer Science and Networks, Kyushu Institute of Technology, Fukuoka Prefecture, Japan

**Keywords:** Sentiment analysis, Stocktwits, Stock price prediction, Machine learning, SVM, SPY, FinBERT, Financial

## Abstract

Investor sentiment plays a crucial role in the stock market, and in recent years, numerous studies have aimed to predict future stock prices by analyzing market sentiment obtained from social media or news. This study investigates the use of investor sentiment from social media, with a focus on Stocktwits, a social media platform for investors. However, using investor sentiment on Stocktwits to predict stock price movements may be challenging due to a lack of user-initiated sentiment data and the limitations of existing sentiment analyzers, which may inaccurately classify neutral comments. To overcome these challenges, this study proposes an alternative approach using FinBERT, a pre-trained language model specifically designed to analyze the sentiment of financial text. This study proposes an ensemble support vector machine for improving the accuracy of stock price movement predictions. Then, it predicts the future movement of SPDR S&P 500 Index Exchange Traded Funds using the rolling window approach to prevent look-ahead bias. Through comparing various techniques for generating sentiment, our results show that using the FinBERT model for sentiment analysis yields the best results, with an F1-score that is 4–5% higher than other techniques. Additionally, the proposed ensemble support vector machine improves the accuracy of stock price movement predictions when compared to the original support vector machine in a series of experiments.

## Introduction

According to the efficient market hypothesis (EMH) (Fama, 1970), the stock market is efficient because it is assumed that all available information has been reflected in stock prices. If we assume that the EMH is true, then the random walk hypothesis (Fama, 1965) states that future news is random and investors cannot use their prior knowledge to generate excess profits. However, numerous studies conducted over the past few decades have shown that it is possible to predict future market prices through techniques such as fundamental analysis, technical analysis, and sentiment analysis. Among them, sentiment-based stock price prediction involves analyzing published news, articles, and social media data to predict future stock prices (Nousi & Tjortjis, 2021). In recent years, an increasing number of investors have been sharing their market perspectives on social media. Therefore, in this study, we aimed to collect use investor opinions from an investor social media site, Stocktwits (https://Stocktwits.com/), combine them with historical stock data, and use a machine learning model to predict future stock price movements.

**Figure 1:**
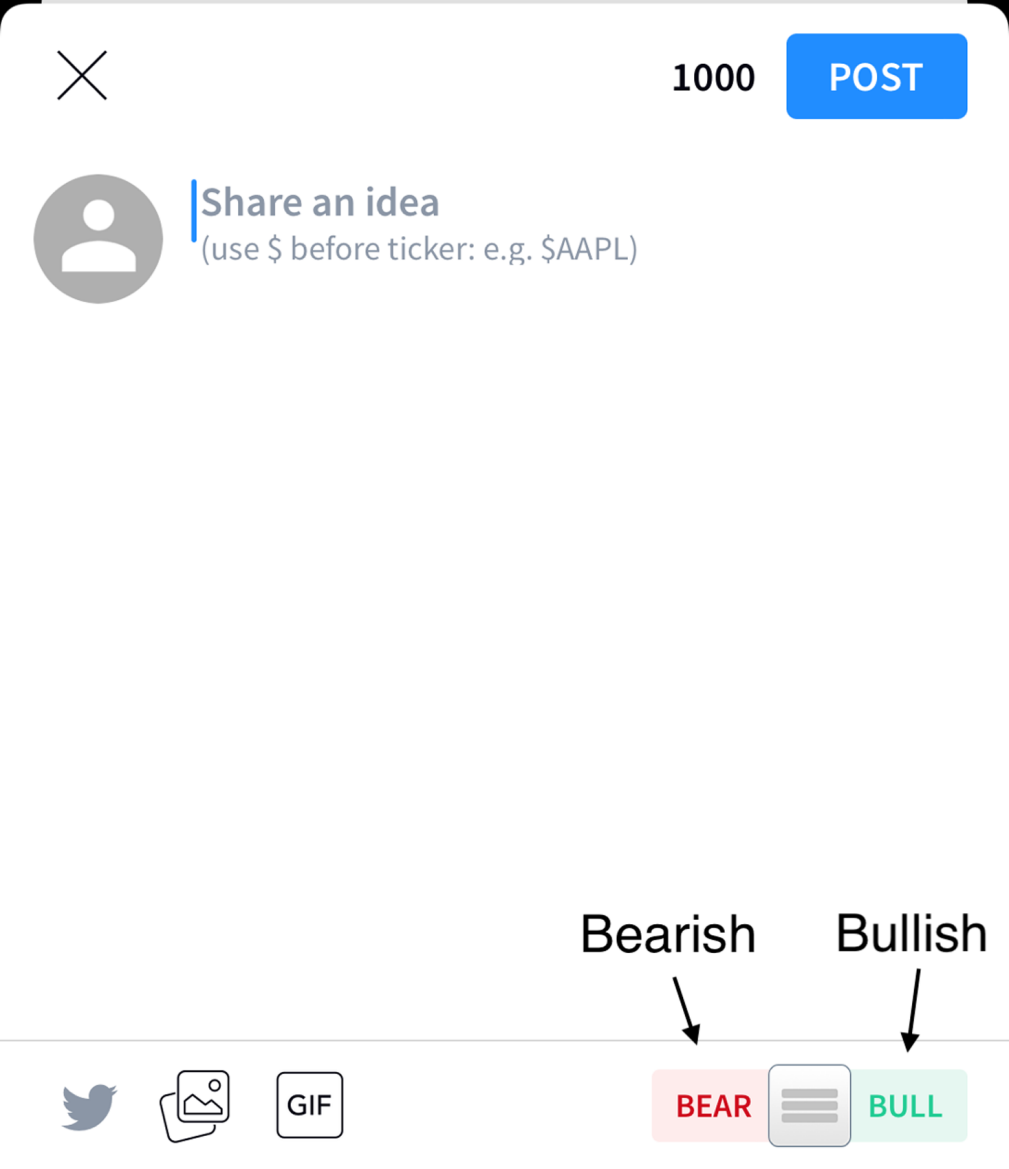
The user interface for providing comments and sentiment about particular stocks.

Stocktwits (https://Stocktwits.com/) is a popular online community for investors and traders, similar to Twitter. Founded in 2008, the website allows users to discuss specific stocks and share their opinions about their future prospects. Through conversations with other investors and professional traders, users can analyze the market and make informed decisions about their investments. Figure 1 shows that the user can provide comment about certain stocks, and the lower right corner allows the user to provide a bullish or bearish sentiment, or to not provide any sentiment.

On Stocktwits, users have the option to label their comments as bullish or bearish, or not to label them. This makes it challenging to predict stock movements by analyzing sentiment on Stocktwits. There are several approaches for collecting investor sentiment. The simplest approach is (1) to collect sentiment only from user-labeled comments. This is because users actively label the sentiment, reducing the risk of sentiment classification errors. However, the main disadvantage is that the sentiment data may be insufficient, resulting in calculated investor sentiment indexes that do not adequately reflect market sentiment. Therefore, most studies use one of the following two methods: (2) use general-purpose sentiment classifiers such as VADAR (Hutto & Gilbert, 2014) or Textblob (https://textblob.readthedocs.io/en/dev/) to analyze all comments (Koukaras, Nousi & Tjortjis, 2022); (3) use all user-labeled comments on Stocktwits to build a sentiment classifier and label comments in order to increase the amount of sentiment (Gupta & Chen, 2020). Nevertheless, the accuracy of sentiment classification won’t be entirely accurate if a sentiment classifier models employed to classify comments. Therefore, it is necessary to consider whether to use a sentiment classifier in predicting future stock price movements.

Due to the fact that Stocktwits only allows users to provide bullish and bearish sentiment labels, this may result in an inaccurate calculation of the sentiment index for two-class sentiment analysis models. This will negatively impact the accuracy of stock forecasts. To improve the accuracy of sentiment indexes for stock price movement prediction, we propose a novel approach: employing FinBERT (Araci, 2019), a state-of-the-art sentiment analysis model in the financial domain, to classify comments on Stocktwits as positive, negative, or neutral, which can significantly reduce noise and thus overcome some limitations of using a two-class sentiment classifier on Stocktwits comments. While FinBERT is often used to analyze financial articles, news, and reports, these data sources are limited and struggle to provide real-time and massive data like social media, which is essential for achieving ideal prediction accuracy. Therefore, we apply FinBERT to classify comments on Stocktwits to increase its macroscopic level of understanding, capturing the overall sentiment of a large number of investors and their views on specific stocks on social media.

As mentioned, the sentiment classifier used in this research is the FinBERT. After sentiment classification, our objective is to predict future stock price movements. In previous studies, support vector machines (SVM) (Cortes & Vapnik, 1995) have been used to predict stock prices with excellent results (Tripathy, 2019). Furthermore, to enhance the accuracy of stock price movement prediction, we propose a novel model that combines the bagging technique (Breiman, 1996) with support vector machines. Few studies in the past have mentioned combining these two techniques to predict stock price movements. In this research, we compare their performance through five experiments.

In addition, most existing studies predict the price movement between the closing price and the opening price. However, this does not provide flexibility for investors who must make immediate or same-day trading decisions. Therefore, we collect the intraday stock price to predict the price change with the opening price.

Based on above, the main contributions of this study are as follows:

1. We conduct sentiment-based stock price movement prediction using FinBERT on a social media, Stocktwits, to raise the macro-level of sentiments and improve accuracy.
2. We employ various techniques to obtain sentiment data and compare each performance in forecasting stock price movements.
3. We use ensemble support vector machine to enhance the accuracy of stock price movement prediction.
4. We change the forecast cut-off time from closing time to intraday time (for example, 12:00 EST) to increase trading flexibility.

The organization of this article is as follows: The next section provides a brief background on sentiment analysis and the FinBERT model, as well as a review of recent studies on sentiment-based stock price movement prediction. The ‘Methodology’ section presents the proposed approach for stock prediction, including data collection and preprocessing, calculation of the sentiment index, and the proposed ensemble support vector machine. The ‘Experimental Setup’ section provides an overview of data preparation, feature extraction, model building, and evaluation metrics. The ‘Results’ section provides details about each experiment and their respective outcomes. The ‘Discussion’ section compares the findings with previous studies. Finally, the ‘Conclusion’ section summarizes the research findings and outlines future directions.

## Related Work

In this section, we describe the background and recent work in sentiment-based stock price movement prediction. This includes sentiment analysis techniques, the use of support vector machine in stock price prediction, recent related studies, and the FinBERT model.

### Sentiment analysis techniques

Sentiment analysis is a subfield of natural language processing (NLP) that can deduce the sentiment or opinion of a phrase or text and classify it as positive, negative, neutral, *etc.*, (Sidogi, Mbuvha & Marwala, 2021). One of the sources for obtaining comments from people is social media, and in recent years, there have been many studies that have used insights from social media comments to solve various problems. Shamoi et al. (2022) used Twitter posts to understand the public’s perception of plant-based diets and employed sentiment analysis techniques to determine if the sentiment in the text was positive or negative. These findings can help medical professionals develop appropriate health plans and encourage more people to maintain healthy plant-based diets. Ko & Chang (2021) conducted sentiment analysis on posts from PPT platform and used long short-term memory (LSTM) model to predict stock prices. However, due to the widespread nature of social media, there is the potential for personal attacks or discrimination based on race and gender to occur on social media. Raman et al. (2022) explained that although many companies have implemented various algorithms on their platforms, it is still difficult to regularly detect such comments, and they continue to appear on the platforms, causing negative impacts on target populations. Therefore, the study compared different hate and attack detection algorithms to detect speech with hateful sentiment. Therefore, the application of sentiment analysis is widely applicable and carries significant commercial value.

### Sentiment-based stock price movement prediction using support vector machine

Support vector machine (SVM) is a technique for supervised learning that was introduced by Cortes & Vapnik (1995) and may be used to address problems related to both regression and classification. SVM may be utilized to achieve high performance in classification tasks, provided that it is equipped with the kernel function. When the data in the initial space cannot be separated by linear classifier, using the kernel function to perform nonlinear projection to separate the data in a higher dimensional space is what the objective of employing the kernel function is. Due to the fact that the stock market is a classic example of a non-linear, dynamic system, SVM is often used in predicting stock prices. Even though SVM does well in solving classification problems, it still has some drawbacks. According to Auria & Moro (2008), one drawback of SVM is the lack of transparency of results, which makes it difficult to get the classified score and probability. This limitation may hinder our ability to make flexible decisions, particularly when buying or selling based on the probability of a classification result.

According to the findings of Hu, Zhu & Tse (2013), SVM has good generalization capacity as well as strong resistance to overfitting difficulties. In contrast, training a SVM model is analogous to solving a linearly constrained quadratic programming problem, and SVM classification always produces a unique global optimal solution. This is in contrast to training a neural network, which requires nonlinear optimization and runs the risk of becoming mired in a local minimum solution. Additionally, the study forecasts stock prices using a combination of annual reports and macroeconomic factors. The final findings demonstrate that SVM is a useful predictive technique for use in stock forecasting.

In a study proposed by Ren, Wu & Liu (2019), it was believed that sentiment features may contain valuable information about the basic value of assets and can be regarded as one of the leading indicators of the stock market. They also mentioned that SVM can solve nonlinear problems by transforming them into quadratic programming and has a unique and globally optimal solution, making it a useful tool for predicting the price movement of the stock market. The researchers implemented fivefold cross validation using SVM, but found that doing so caused look-ahead bias. To eliminate this bias, they integrated SVM with the realistic rolling window approach. In order to avoid overfitting in our research, the rolling window method will be used to verify its effectiveness.

### Using Stocktwits comments to make sentiment-based stock price movement predictions

In a study conducted by Koukaras, Nousi & Tjortjis (2022), comments on stocks were collected from people on Twitter and Stocktwits, and stock price movements were predicted through sentiment analysis and machine learning. The study used VADER as a sentiment analyzer and predicted the movement of Microsoft’s (MSFT) stock price. Various machine learning models were tried for predicting stock price movements, and the final results showed that SVM outperformed the other classification models. This study used sentiment analysis in social media for stock forecasting in the US market, achieving good results and inspiring the researchers to use SVM to solve the problem of sentiment-based stock price movement prediction in their own study.

Gupta & Chen (2020) collected comment data from Stocktwits between January 1st, 2019 and September 30th, 2019. They developed several sentiment analyzers for different stocks using techniques such as logistic regression and TF-IDF. Then, they experimented with several US stocks for stock price movement prediction and found that adding more sentiment data helped improve the accuracy of predicting stock price movement.

Furthermore, in recent years, many studies have developed two-class sentiment analysis models using comments on Stocktwits. This is beneficial for our research and comparison. Lee, Gao & Tsai (2020) fine-tuned the pre-trained BERT on a labeled sentiment dataset and achieved an accuracy of over 87.3% in recognizing the sentiment of investors. Bozanta et al. (2021) conducted multiple experiments and ultimately used RoBERTa (Liu et al., 2019) to achieve an accuracy of 83–88%. Cao et al. (2022) fine-tuned the roberta-base model on 3.2 million comments from Stocktwits, with user-labeled tags of bullish or bearish, and achieved a validation accuracy of 93.43%. Therefore, our study uses the model proposed by Cao et al. (2022) for two-class sentiment analysis to predict stock price movements and compares it with the method proposed in this article.

### FinBERT: financial sentiment analysis with BERT

**Figure 2:**
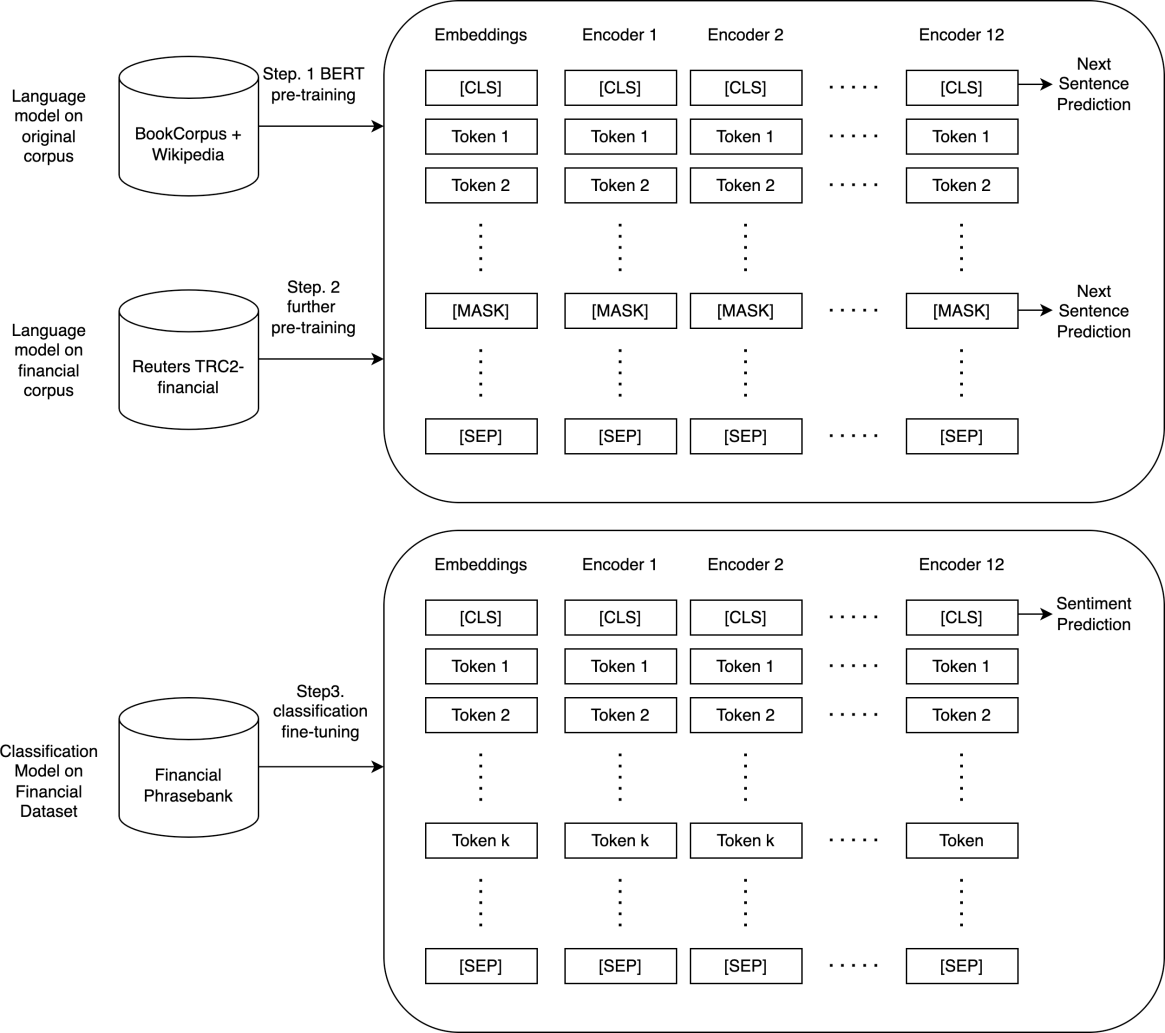
Overview of pre-training, further pre-training, classification, and fine-tuning of FinBERT.

FinBERT (Araci, 2019) is the first application of BERT (Devlin et al., 2018) in the financial domain, which is a pre-trained sentiment analysis model specialized in analyzing financial texts. FinBERT first pre-trains the BERT language model using a large financial dataset and then fine-tunes it on a specific financial dataset (https://huggingface.co/ProsusAI/finbert). The SOTA (state-of-the-art) level is achieved on the Financial Phrasebank dataset. The dataset consists of 4,840 sentences from English language financial news categorized by sentiment (Malo et al., 2013), and the accuracy is 15% higher than the current state-of-the-art model. The model can produce three classifications of sentiment, including positive, negative, and neutral, enabling us to detect neutral sentiment and compute the sentiment index more precisely, thereby enhancing the accuracy of the stock price movement prediction. FinBERT for sentiment analysis often analyzes financial news and articles. FinBERT performs sentiment classification by adding a dense layer after the last hidden state of the [CLS] token, which is the recommended method for using BERT for any classification task. The classifier network is then trained on a labeled sentiment dataset. The procedure is summarized in Fig. 2.

Fazlija & Harder (2022), used the FinBERT model to classify news, forecast the trend of stock prices with the random forest classifier, and measure the investment performance of the models. The performance of the proposed method in the study outperformed the S&P 500 index. However, although this article uses K-fold cross-validation to find the best model parameters, splits the training and test data sets into multiple copies, and trains multiple models for validation, the authors did not mention the use of the rolling window approach in their study. Therefore, in our research, we intend to apply FinBERT to analyze comments on social media, allowing us to gather more opinions about the target asset. Then, by making predictions using the rolling window approach, we can not only avoid the problem of look-ahead bias, but also update the training data over time, which can lead to more accurate predictions.

In this study, we propose using FinBERT to perform sentiment analysis on investors’ comments on Stocktwits in order to predict stock price movements. Few studies have used FinBERT for sentiment analysis on social media. However, we believe that applying it specifically to analyze Stocktwits comments, rather than using a two-class sentiment analysis model, will improve the macro-level sentiment index and enhance the accuracy of forecasting stock price movements.

## Methodology

In this section, we discuss the various sources of data that can be used to analyze stocks, such as historical data, comments, and sentiment data. We also outline the preprocessing steps necessary to analyze comments, including how to calculate a sentiment index. Finally, we introduce a prediction model for stock price movements using ensemble techniques and support vector machines.

### Data collection

**Figure 3:**
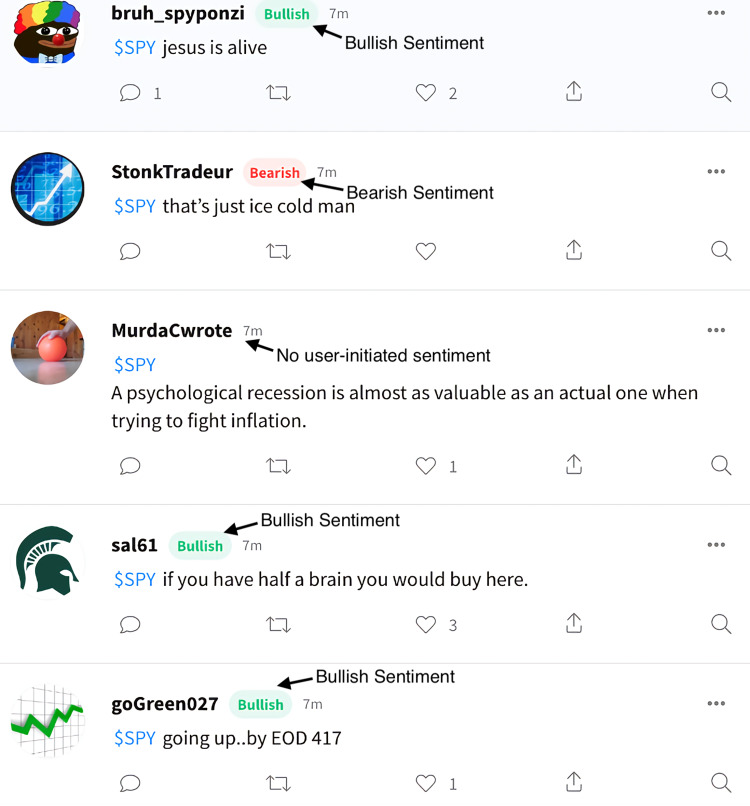
Tweets posted by users on Stocktwits. Users can fill in a text and label it with their view of the future (optional). As you can see in the figure, some tweets have a bullish or bearish sentiment, while others are not label.

On Stocktwits, users may share a message and label it as “bullish” or “bearish”, or they might ignore these sentiments. There are three data columns: message, date/time, and sentiment (optional). The Stocktwits tweets are shown in Fig. 3.

Accessing https://api.Stocktwits.com/ allows us to examine JSON-formatted investor comments on a certain stock from Stocktwits. To get data from Stocktwits, we developed a Node.js app that automatically downloads and saves CSV files with all comments on a specific stock over a certain period. Then, we brought the data into the Python environment to perform more analysis.

The historical stock data is collected from cnYES (https://www.cnyes.com/), and the data columns include stock opening price, closing price, high price, low price, VWAP (volume-weighted average price), volume, trade value, change, and percentage change, as shown in Table 1.

**Table 1:** Historical stock data.

**Date**	**Open**	**High**	**Low**	**Close**	**VWAP**	**Volume**	**Percentage** **change**	**Change**	**Trade value**
2021-12-14	461.4655	464.1062	458.6354	461.7345	460.7819	97109342	−0.688	−3.1987	44958834200
2021-12-13	468.5406	468.9093	464.6343	464.9333	465.9666	87575650	−0.886	−4.1554	40970340100
2021-12-10	467.5839	469.2481	464.8735	469.0886	467.3978	76938003	0.941	4.3746	36065910500
2021-12-09	466.5077	467.9816	464.5048	464.714	465.9068	61177455	−0.675	−3.1589	29141130600
2021-12-08	467.0558	468.3512	465.1924	467.8729	466.852	72113171	0.265	1.2356	33847165200
2021-12-07	462.7808	467.2352	457.0456	466.6373	465.8307	94488145	2.068	9.4567	44082244300

We used the AlphaVantage API (https://www.alphavantage.co/) to obtain historical real-time stock prices. This enables us to acquire a minute-by-minute view of stock prices, allowing us to make more flexible forecasts. However, the data is restricted to the last two years. Table 2 shows historical stock price data in 15-minute increments.

**Table 2:** Samples of historical stock real-time data.

**Time**	**Open**	**High**	**Low**	**Close**	**Volume**
2021-03-26 20:00:00	390.5090074	390.5484847	390.3609677	390.4596608	25556
2021-03-26 19:45:00	390.3214904	390.5090074	390.2820132	390.5090074	21009
2021-03-26 19:30:00	390.3807063	390.3807063	390.2425359	390.2524052	6795
2021-03-26 19:15:00	390.3313598	390.3905756	390.2721439	390.2918825	11586
2021-03-26 19:00:00	390.3510984	390.3510984	390.2820132	390.3313598	11730
2021-03-26 18:45:00	390.4300529	390.4300529	390.2425359	390.3510984	21191

### Sentiment data preprocessing

We refer to Cao et al. (2022). Before conducting sentiment analysis, the following text preprocessing steps should be performed:

1. Remove all web links from the text, including those that start with “http://”, “https://”, or “www.”.
2. Replace the HTML character for single quotation marks with standard single quotation marks.
3. Identify and replace hashtags (#) and cashtags ($) in the text. Find all words that start with ”#” or ”$”, such as $AAPL, and substitute them with terms like “cashtag_AAPL”. This change is made to preserve the original symbol information while making it more suitable for text processing.
4. Replace Twitter usernames with a term that begins with “mention_”, followed by the username. This modification is also intended to preserve the original information while rendering it more suitable for text processing.
5. Convert all emojis into their corresponding text descriptions to maintain the emotional or semantic information they convey, but in a format that is easier for the model to understand.

### Stock price movement prediction

**Figure 4:**
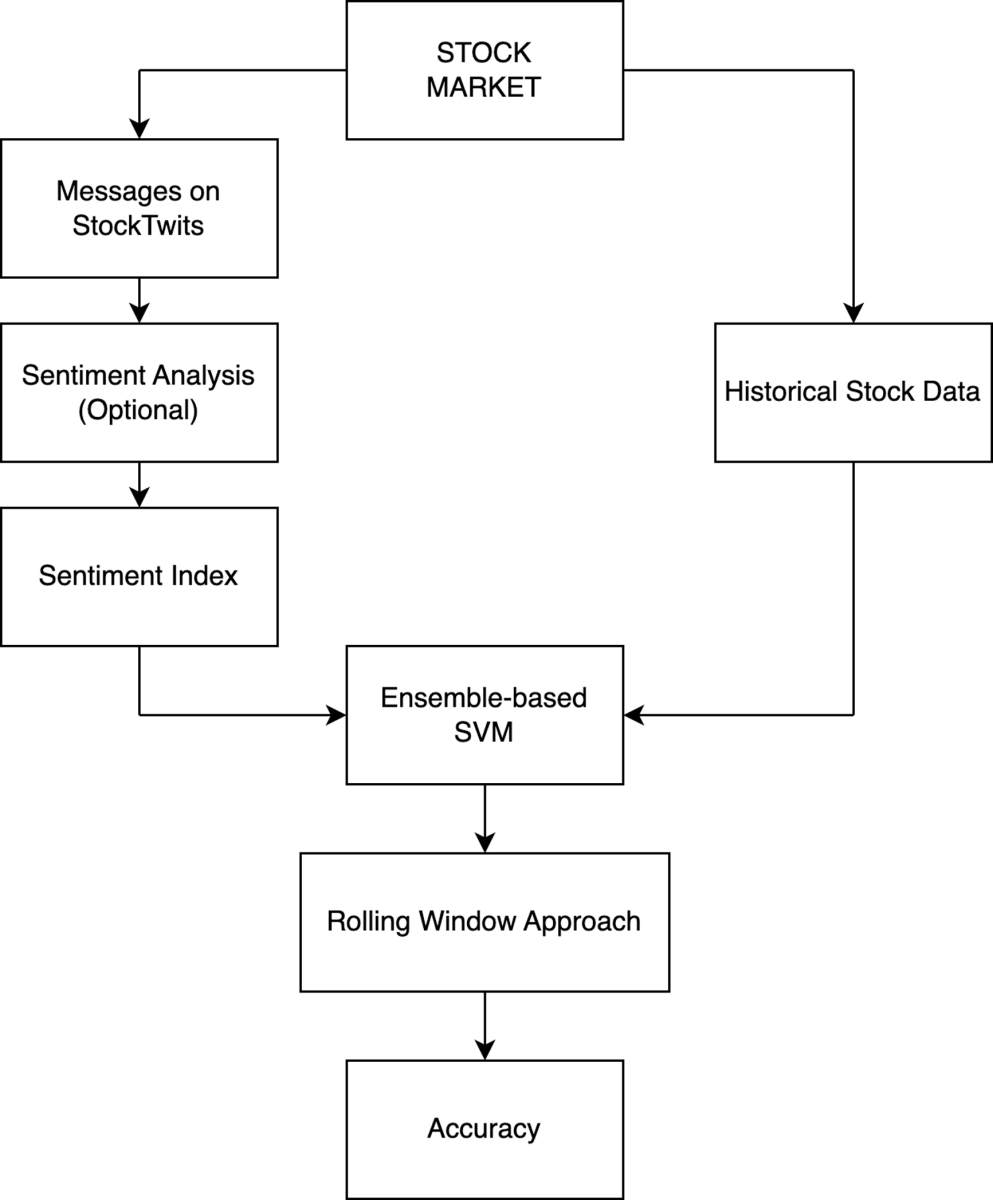
Overview of stock market movement prediction architecture.

The architecture depicted in Fig. 4 was used to predict stock movements. We collected the necessary data, which was divided into two parts: the first part consists of comments about specific stocks on Stocktwits, some of which are not labeled as bullish or bearish, while the second part consists of historical data on stock prices. We can choose to use a two-class sentiment analysis model to label the unlabeled comments or use a three-class sentiment analysis model like FinBERT or VADER to label all comments. Finally, we applied the sentiment index formula to calculate the entity index of comments from multiple periods. To predict stock price movements, we combined the sentiment indexes with historical stock data as features and input them into a prediction model designed for stock price movements. We used the rolling window approach to predict and analyze the accuracy of stock movement predictions.

### Sentiment index calculation

The two-class sentiment index calculation used in this article is related to Antweiler & Frank (2004) methods for calculating sentiment indexes.

\begin{eqnarray*}{S}_{t}=\ln \nolimits \left[ \frac{1+{M}_{t}^{pos}}{1+{M}_{t}^{neg}} \right] . \end{eqnarray*}

This index enables us to compute the sentiment for a period, where ${\mathbi{M}}_{\mathbi{t}}^{\mathbi{pos}}$, ${\mathbi{M}}_{\mathbi{t}}^{\mathbi{neg}}$ are the number of positive and negative messages during that period. If the sentiment index is positive, market sentiment is optimistic. If the sentiment index is negative, market sentiment is pessimistic. If the sentiment index is zero, market sentiment is neutral. This index helps us to determine investor sentiment over a certain time period and also adjusts for overly optimistic or pessimistic signals during calculation.

For three-class sentiment analysis models (including positive, negative and neutral sentiments), refer to the equation used to calculate the sentiment index in Hiew et al. (2019).

\begin{eqnarray*}{S}_{t}= \frac{{M}_{t}^{pos}-{M}_{t}^{neg}}{{M}_{t}^{pos}+{M}_{t}^{neu}+{M}_{t}^{neg}} \end{eqnarray*}

where ${\mathbi{M}}_{\mathbi{t}}^{\mathbi{pos}}$, ${\mathbi{M}}_{\mathbi{t}}^{\mathbi{neg}}$, ${\mathbi{M}}_{\mathbi{t}}^{\mathbi{neu}}$ are the number of positive, negative and neutral messages within the period.

### Support vector machine

**Figure 5:**
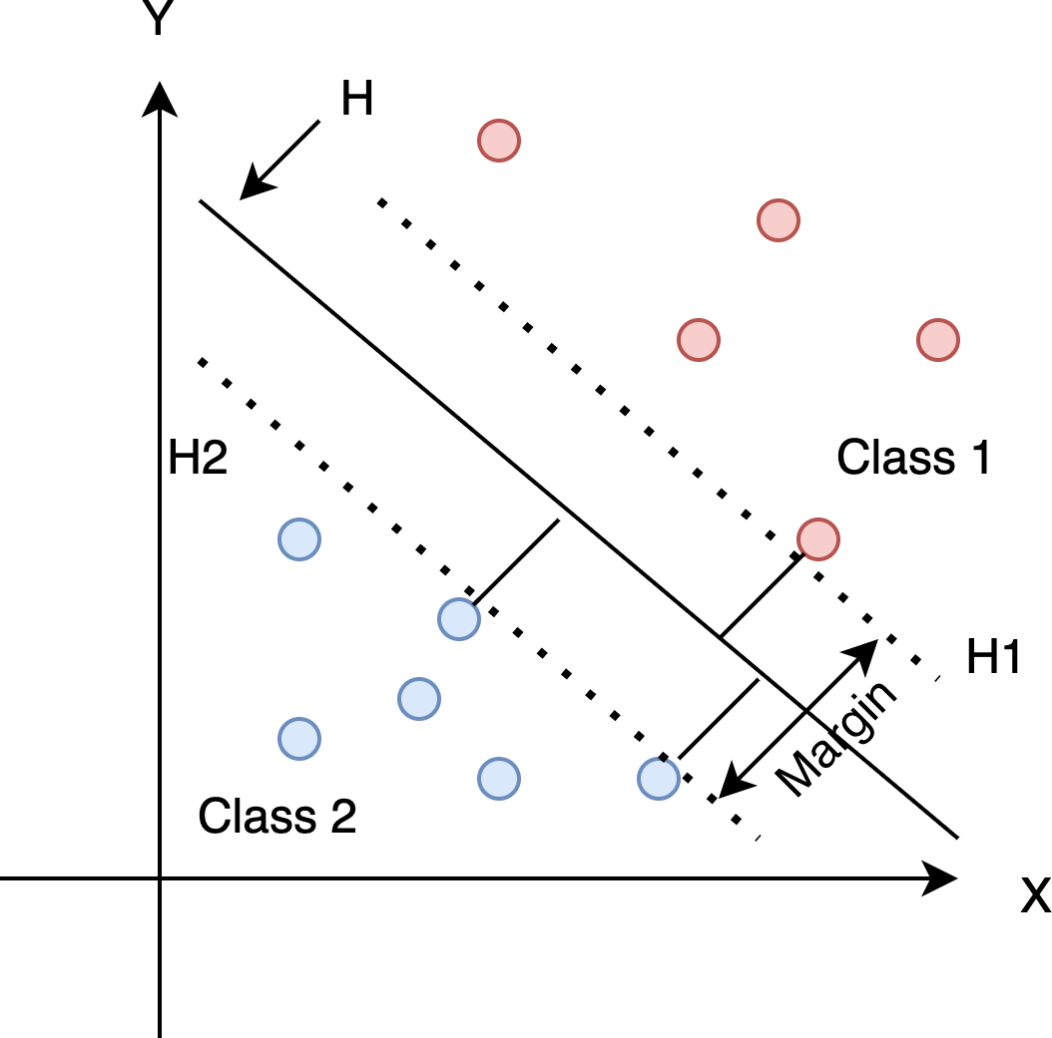
Find the optimum hyperplane that separates the dataset into two categories.

Support Vector Machine (SVM) is a supervised machine learning algorithm developed by Cortes & Vapnik (1995), which can be used to solve classification and regression problems. The concept is simple: find a decision boundary that maximizes the margin between two classes, as shown in Fig. 5.

The equation for the distance from ***x***, ***y*** to ***Ax*** + ***By*** + ***C*** = ***0*** is given by

\begin{eqnarray*} \frac{Ax+By+C}{\sqrt{{A}^{2}+{B}^{2}}} . \end{eqnarray*}

Extending it to the n-dimensional space, we express it as ***w***^***T***^***x*** + ***b*** = ***0***, and the distance as

\begin{eqnarray*} \frac{({w}^{T}x+b)}{{|} \left\vert w \right\vert {|}} \end{eqnarray*}

where w denotes the weight of each feature, b denotes intercept.

If we disregard the issue of mixed data, *i.e.,* if all data can be perfectly classified, we call this hard-margin SVM and divide the two classes according to the equation below.

\begin{eqnarray*} \left\{ \begin{array}{@{}l@{}} \displaystyle {w}^{T}{x}^{ \left( i \right) }+b\geq 1,~\forall {y}^{ \left( i \right) }=1 \\ \displaystyle {w}^{T}{x}^{ \left( i \right) }+b\leq -1,~\forall {y}^{ \left( i \right) }=-1  \end{array}. \right. \end{eqnarray*}

And simplify the formula as follows

\begin{eqnarray*}{y}^{ \left( i \right) } \left( {w}^{T}{x}^{ \left( i \right) }+b \right) \geq 1, \forall i=1,\ldots ,n. \end{eqnarray*}

The solution to SVM is a simple optimization problem. Under some conditions, the larger the margin distance between the two classes, the better. The formula is as follows

\begin{eqnarray*}\min \nolimits \frac{1}{2} {|}{|}w{|}{{|}}^{2} \end{eqnarray*}

\begin{eqnarray*}s.t. {y}_{i} \left( {w}^{T}{x}_{i}+b \right) \geq 0, \forall i=1,\ldots ,n. \end{eqnarray*}

However, it is rare that real-world data would include examples that can be precisely identified. Therefore, we allow certain points to transcend the initial decision limit and enable some unusual samples to be wrongly classified. Although this may result in incorrect categorization, the generalization capacity may be superior in practical applications. This kind of SVM is known as soft-margin SVM, using the following formula


\begin{eqnarray*}\min _{w,\mathit{\xi i}} \frac{1}{2} {w}^{T}w+C\sum _{i=1}^{n}\xi i \end{eqnarray*}

$s.t. {y}_{i} \left( {w}^{T}{x}_{i}+b \right) \geq 1-{\xi }_{i},{\xi }_{i}\geq 0,\forall $i =1 , …, *n*

where **ξi** is a tolerable training error, and C determines how much penalty weight is to be given.

From the above equation, we can see that if C is larger, the space for error tolerance is smaller, and if C is positive infinity, it will become a hard-margin SVM. Conversely, if C is smaller, the space for error tolerance is larger.

To solve the optimal solution of the primal problem, we can transform it into its dual problem, as shown in the following equation

\begin{eqnarray*}\max _{\alpha }\sum _{i=1}^{n}{\alpha }_{i}- \frac{1}{2} \sum _{i=1}^{n}\sum _{j=1}^{n}{\alpha }_{i}{\alpha }_{j}{y}_{i}{y}_{j}K({x}_{i},{x}_{j}) \end{eqnarray*}

s.t. ${\mathop{\sum }\nolimits }_{i=1}^{n}{\alpha }_{i}{y}_{i}=0$

\begin{eqnarray*}0\leq {\alpha }_{i}\leq C,\forall i=1,\ldots ,n \end{eqnarray*}

where $\alpha ={ \left( {\alpha }_{1},\ldots ,{\alpha }_{\mathbi{n}} \right) }^{\mathbi{T}}$ is a Lagrange multiplier, and $\mathbi{K} \left( {\mathbi{x}}_{\mathbi{i}},{\mathbi{x}}_{\mathbi{j}} \right) $ signifies that we employ a kernel function to redefine the inner product of ***x***_***i***_ and ***x***_***j***_. There are several common kernel functions shown in Table 3.

**Table 3:** Common Kernel functions.

**Name**	**Kernel function**
Linear	${K}_{linear} \left( {x}_{i},{x}_{j} \right) ={x}_{i}\cdot {x}_{j}$
Polynomial	${K}_{poly} \left( {x}_{i},{x}_{j} \right) ={ \left( \left( {x}_{i}\cdot {x}_{j} \right) +c \right) }^{d}$
RBF	${K}_{rbf} \left( {x}_{i},{x}_{j} \right) ={e}^{-\gamma ({x}_{i}-{x}_{j})^{2}}$

Finally, the decision function $\mathbi{D} \left( \mathbi{x} \right) =\mathbi{sgn}({\mathbi{w}}^{\ast }\cdot \mathbi{x}+{\mathbi{b}}^{\ast })$ is given by

\begin{eqnarray*}D \left( x \right) & =sgn(\sum _{i=1}^{n}{y}_{i}{\alpha }_{i}\ast K \left( {x}_{i},{x}_{j} \right) +{b}^{\ast }) \end{eqnarray*}

\begin{eqnarray*}{b}^{\ast }& ={y}_{j}-\sum _{i=1}^{n}{y}_{i}{\alpha }_{i}\ast K({x}_{i},{x}_{j}). \end{eqnarray*}

### Ensemble-based technique

According to Mohareb et al. (2016), the ensemble-based technique is based on the development of several classifiers, with the final prediction output being a composite of all classification results in the integration. Suppose we use n classifiers *ɛ*  = {** *C***_1_, …, ***C***_***n***_} for predicting a sample, if the result of ***C***_1_ is wrong, but the results of ***C***_2_, …, ***Cn*** are correct, then the result can be classified correctly by majority voting.

Individual classifiers must have good accuracy for this method to be applicable. If there are a total of T classifiers for a c-class problem, the ensemble choice will be correct if at least [***T***/***c*** + 1] classifiers select the correct class. Assume that each classifier of *ɛ* has a probability p of selecting the correct class. The ensemble’s chance of picking the right class has a binomial distribution, and the probability of picking ***k*** > ***T***/***c*** + 1 correct classifiers out of ***T*** is calculated as follows: (Mohareb et al., 2016).

\begin{eqnarray*}{\mathbi{p}}_{}=\sum _{\mathbi{k}= \left( \frac{\mathbi{T}}{\mathbi{C}} \right) +1}^{\mathbi{T}} \left( \frac{\mathbi{T}}{\mathbi{k}} \right) {\mathbi{p}}^{\mathbi{k}}(1-\mathbi{p})^{(\mathbi{T}-\mathbi{k})}. \end{eqnarray*}

### SVM ensemble with bagging

According to Kim et al. (2002) in bagging, several SVMs are individually trained using the bootstrap approach and then aggregated using a suitable combination technique. The training set needs to be split into L subsets to construct the SVM ensemble with L independent SVMs. Based on statistical evidence, it is necessary to make the training sets as diverse as feasible to get a greater improvement in the aggregation result. We employ the bootstrap method to do this.

**Figure 6:**
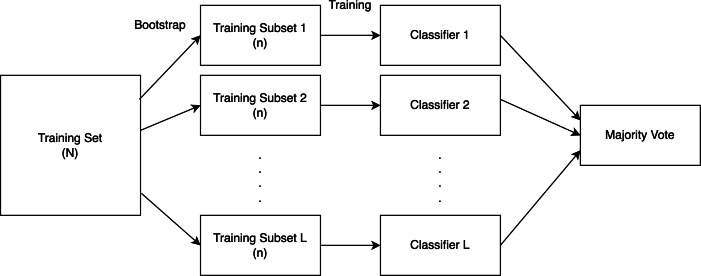
A diagrammatic representation of the bagging procedure.

The bootstrap method was proposed by Efron (1979). In simple terms, each model is trained by randomly picking n sample data from the training set, and each sample is placed back after picking, as shown in Fig. 6. We will utilize models with trained subsets to forecast the test data, and the final prediction will be generated using these models through a majority vote, as explained below.

Majority vote is the simplest method for combining classifiers. The results of many classifiers are mixed by majority vote. The output with the most votes is then selected as the final classification decision (Kim & Upneja, 2021).

## Experimental Setup

This section presents the experimental setup, including data preparation, feature extraction, model building, and evaluation metrics.

### Data preparation

**Figure 7:**
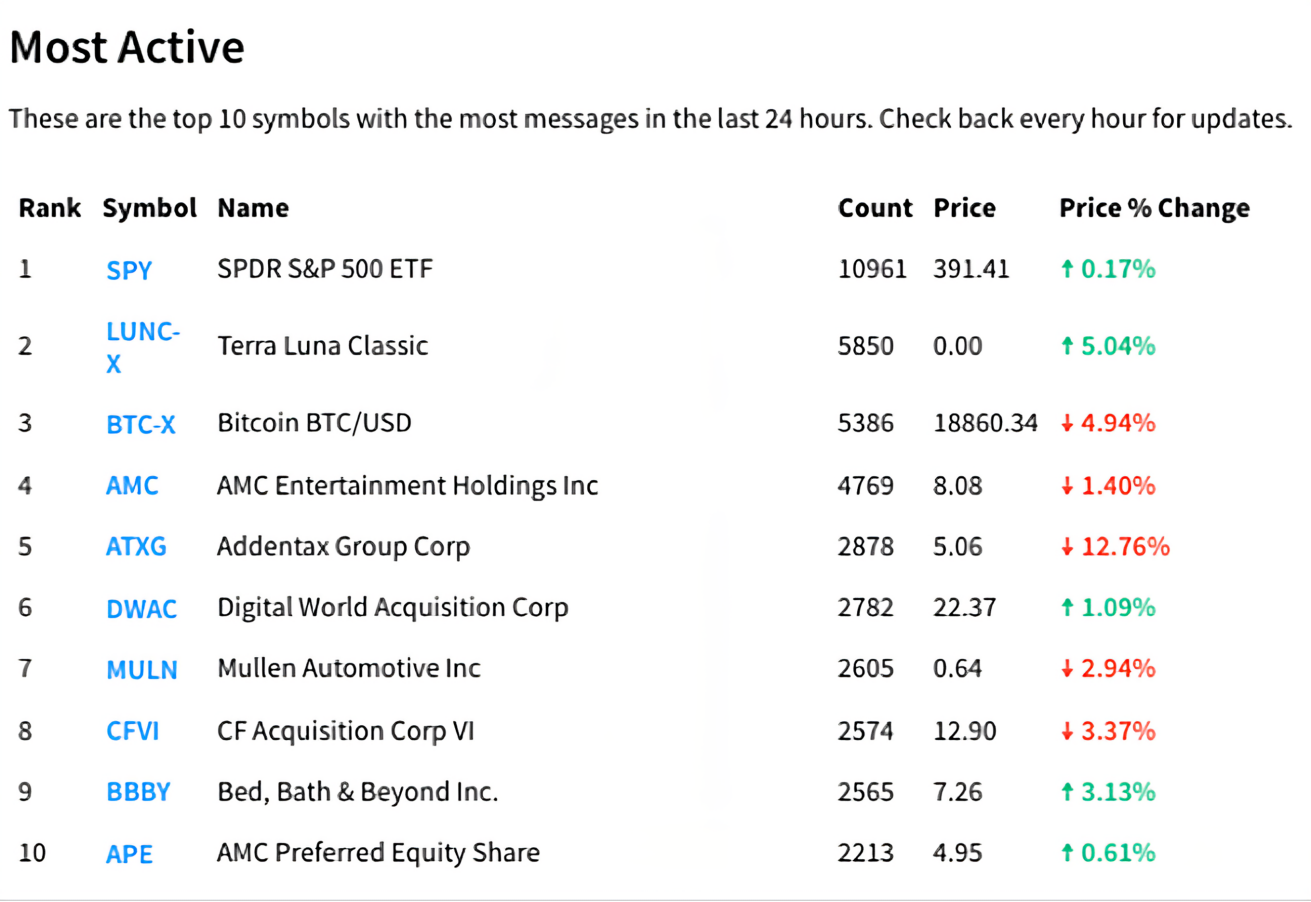
Top 10 symbols with the most messages in September 7, 2022.

The SPDR S&P 500 Index ETF (SPY) was used in this study because it is currently the most popular S&P 500 ETF (Exchange Traded Fund) and one of the stocks with the most Stocktwits discussions. As shown in Fig. 7, this is the most active rank at Stocktwits on September 7, 2022. As we can see, SPY has significantly more discussions than any other stock, and based on our observations, SPY has been in the top 3 for an extended period of time.

 We collect all SPY comments and historical stock data on Stocktwits from April 1, 2020, to February 28, 2022, a total of 482 trading days, of which approximately 1.33 million Stocktwits messages were labeled and 1.73 million messages were not labeled by users, for a grand total of 3.06 million messages. The training set starts on April 1, 2020, and the period from April 05, 2021, to February 28, 2022 is used as test data.

### Feature extraction

We train the model using a total of 10 features, as shown in Table 4, including three sentiment features and seven derived from the historical stock data. We have found that considering both the pre-market sentiment and the after-market sentiment from the day before may significantly increase the accuracy of our stock price movement forecast. This is due to the fact that the stock market places a significant emphasis on these two time periods.

**Table 4:** Extracted features.

**Feature**	**Description**
S1	The sentiment index of the predicted day’s pre-market trading session
S2	The sentiment index during after-hours trading on the day before the predicted day
S3	The sentiment index during intraday trading on the day before the predicted day
S4	The opening price on the trading day before the predicted day
S5	The closing price on the trading day before the predicted day
S6	The highest price on the trading day before the predicted day
S7	The lowest price on the trading day before the predicted day
S8	The volume on the trading day before the predicted day
S9	The intraday percentage change on the trading day before the predicted day
S10	The total transaction amount on the trading day before the predicted day

The calculation formulas for features ***S***1 to ***S***3 are shown in the subsection named ‘Sentiment Index Calculation’ of previous section. To obtain feature values, all comments about a certain stock on Stocktwits during the specified period must be obtained and processed through a sentiment analysis model. Then, the formulas for calculating the sentiment index can be used to compute these feature values. Features ***S***4 to ***S***10 are historical stock data and do not require calculation.

The features we have selected are accessible prior to the opening of the forecast day, which allows us to obtain sentiment and make forecasts without overlap. This means that the forecast results will be available to investors at the start of the market, enabling informed trading decisions.

### Model building

Since we are forecasting a moving price trend (either upward or downward) rather than the actual price of the SPDR S&P 500 Index ETF, we have to convert a regression problem into a categorical one. This is because we are focusing on moving price trends rather than actual prices. It is necessary for us to label the data based on the equation that is shown below.

\begin{eqnarray*}y= \left\{ \begin{array}{@{}l@{}} \displaystyle 1,~~Price~at~12:00~EST~\geq ~Opening~Price \\ \displaystyle 0,~~Price~at~12:00~EST~\lt ~Opening~Price  \end{array}. \right. \end{eqnarray*}

We used a support vector machine (SVM) ensemble with bagging to build the model. This required setting the parameters for both the support vector classifier (SVC) and the bagging classifier. The SVC has several important hyperparameters, such as the kernel function, C (the regularization parameter), and gamma (the kernel coefficient). These parameters are shown in detail in Table 5. We also used the BaggingClassifier from the scikit-learn python library (Pedregosa et al., 2011). The bagging classifier has several important hyperparameters that must be set, such as the base estimator, the number of estimators (n_estimators), and whether samples are drawn with replacement (bootstrap). These parameters are shown in detail in Table 6.

### Evaluation metrics

The equation for the classification accuracy is as follows, which can represent the percentage of the total number of correct predicted results.

\begin{eqnarray*}Accuracy= \frac{TP+TN}{TP+FP+FN+TN} \end{eqnarray*}

where TP denotes that the predicted value is 1 and the true value is also 1; TN denotes that the predicted value is 0 and the true value is also 0; FP denotes that the predicted value is 1 but the true value is 0; and FN denotes that the predicted value is 0 but the true value is 1.

\begin{eqnarray*}Precision= \frac{TP}{TP+FP} \end{eqnarray*}

where precision is the percentage of times out of a sample of predicted stock price increases that the actual price went up.

\begin{eqnarray*}Recall= \frac{TP}{TP+FN} \end{eqnarray*}

where recall is the percentage of times out of a sample of actual stock price increases that the predicted price went up.

**Table 5:** Experimental settings of the Support Vector Classifier.

**Parameter**	**Value**
C	1
kernel	rbf (radial basis function)
*γ* (gamma)	scale

**Table 6:** Experimental settings of the Bagging Classifier.

**Parameter**	**Value**
base_estimator	SVC (Support Vector Classifier)
n_estimators	100
Bootstrap	True

In predicting whether a stock price will rise, precision is considered more important than accuracy. In a bull market, both precision and recall are equally important for decision-making. However, in a bear market, recall becomes more crucial than precision as it has a greater impact on potential financial loss (http://www.bituzi.com/2019/10/confusionmatrix.html).

\begin{eqnarray*}F1~Score= \frac{2~Precision\ast Recall}{Precision+Recall} . \end{eqnarray*}

The F1-score above is the harmonic mean of precision and recall. A score of 1 represents the highest performance, and a score of 0 represents the worst. Both precision and recall contribute equally to the F1-score; hence, the closer the score is to 1, the better the model (https://scikit-learn.org/stable/modules/generated/sklearn.metrics.f1_score.html). Therefore, for this topic, we evaluate the methods using the F1-score.

## Results

In this study, we conducted five experiments to measure the performance of incorporating sentiment indexes in predicting stock price movements. In addition, through these experiments, we can determine whether the addition of sentiments can help the model in forecasting stock price movements and compare the performance of various methods of obtaining sentiment indexes. The experiments are as follows

Exp. 1.: In building the model, only historical stock data was utilized as features and no sentiment data was taken into account. So, only features ***S***4 − ***S***10 from Table 4 were employed in the model.
Exp. 2.: In addition to using historical stock data, we include sentiment indexes calculated from sentiment data provided by users as features.
Exp. 3.: In order to increase the overall quantity of sentiments, not only user-provided sentiments but also unlabeled comments are classified to be bullish or bearish using a binary classification sentiment analysis model. We employ the roberta-base-Stocktwits-finetuned (https://huggingface.co/zhayunduo/roberta-base-Stocktwits-finetuned) sentiment analysis model in this experiment, which is a sentiment analysis model trained on Stocktwits comments and can output bullish or bearish with 93.43% accuracy.
Exp. 4.: All comments are labeled by the FinBERT model, and sentiment indexes are calculated as features.
Exp. 5.: All comments are labeled by the VADER model, and sentiment indexes are calculated as features as Koukaras, Nousi & Tjortjis (2022) did in their similar experiment using VADER. The model also outputs three classes.

**Figure 8:**
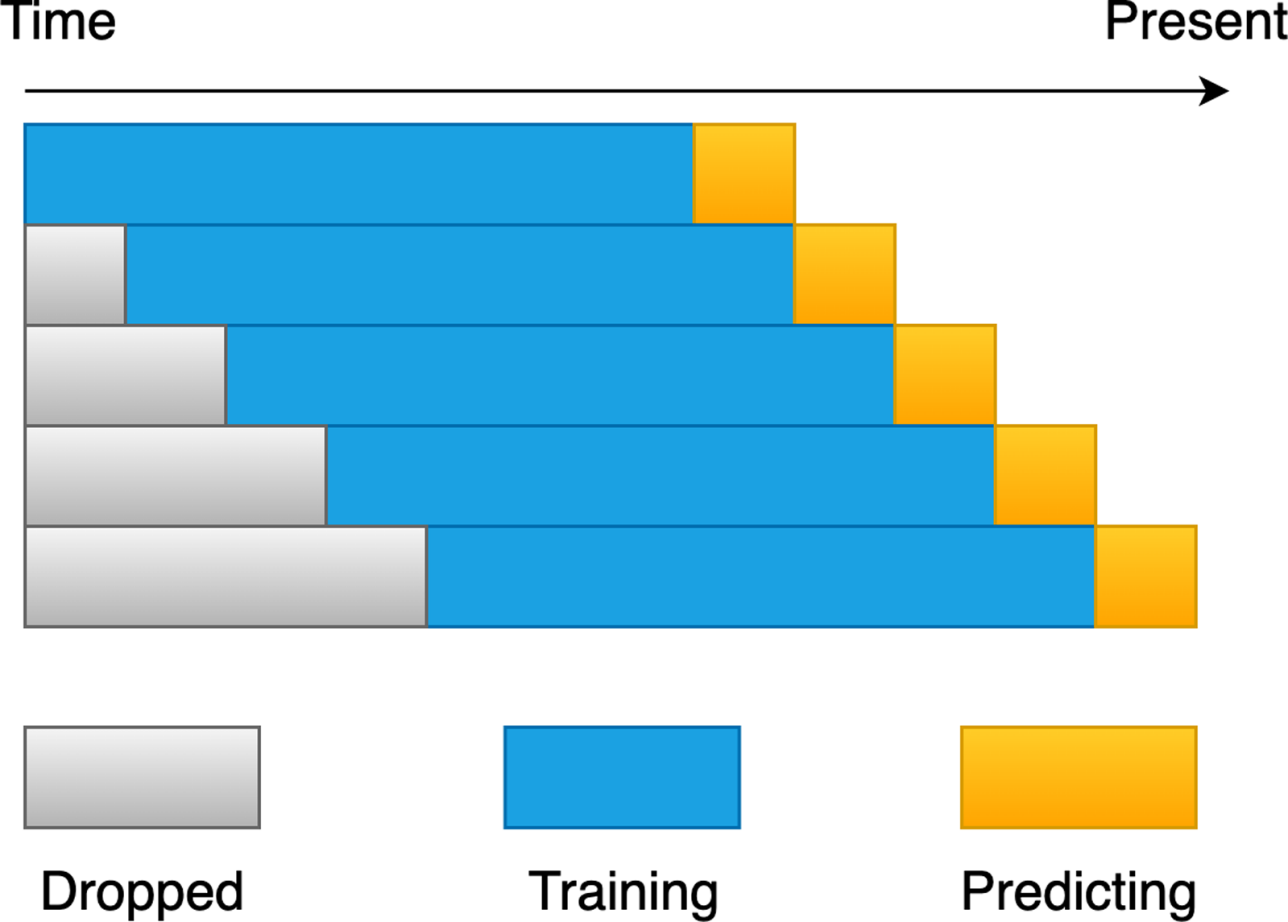
A diagrammatic representation of the rolling window procedure.

According to Ren, Wu & Liu (2019), randomly selecting the training and test sets in our dataset may result in a look-ahead bias. For instance, we predict today’s stock price movement, but our training set contains data from tomorrow and the next day, giving the classification model a head-start on future trends that cannot be achieved in real-world applications. We solve the problem of look-ahead bias by using the rolling window approach. Figure 8 illustrates the idea in its entirety. To begin, it is necessary to determine the rolling window size that is most suitable for each experiment. The size was determined by training on previous days, forecasting the movement of the following day until the end of the dataset, and picking the window size that had the greatest accuracy for the experiment. When we have chosen the optimal size for the rolling window, we can then use this size into the future in order to forecast the price movement of the new day. As a result, we train and validate the model using the rolling window approach, which allows us to effectively avoid the issue of look-ahead bias and prevent the model from overfitting the data.

In addition, we believe that the size of the rolling window should not be too small, as market conditions may change in the short term. For example, if the market is currently bullish, certain factors or market news may cause it to turn bearish. If the data used to train the model is not sufficient, even if the prediction results are good, the generalization ability of the model may be worse. Therefore, although the dataset for this research is limited, we set the rolling window training size to be between 232 and 252 (about one year’s worth of working days), the test size of a window is 1 for experimentation and analysis, and the training window size is set to a number that is divisible by 2. So, we train 11 prediction models with different training window sizes in each experiment. Furthermore, due to the different market conditions between bull and bear markets, the training dataset may be imbalanced. Therefore, we use SMOTE (Chawla et al., 2002), an oversampling technique, to balance the training data.

The best F1-score obtained from the experimental results is presented in Table 7.

**Table 7:** The results of forecasting price movement.

	Original SVM		Ensemble SVM		
	F1-score	Training size in window	F1-score	Training size in window	
Exp. 1	53.57%	236, 238	55.02%	240	
Exp. 2	59.74%	246	61.41%	234	
Exp. 3	58.72%	234	60.16%	234	
Exp. 4	62.19%	236	65.30% (Proposed Method)	238	
Exp. 5	61.04%	238	61.66%	232	

In Exp. 1, the absence of sentiment indexes resulted in less accurate result compared to the other experiments. Therefore, we use the result of Exp. 1 as a baseline for the remaining experiments.

In Exp. 2, the inclusion of actively indicated sentiments from users significantly improves the performance of stock price movement predictions. This demonstrates a strong correlation between investor sentiment and stock price movement.

In Exp. 3, we employed a two-class sentiment analysis model to enhance the overall quantity of sentiments in our analysis. However, we discovered that some of the non-user-labeled comments discussed criticism of politics or platform operations. These were not suitable for classification as bullish or bearish by the model, but the model still assigned a label due to its limitation of outputting only two types of sentiments. As a result, the F1-score of the stock price forecast was lower than in Exp. 2. To further investigate this issue, we randomly selected 100 unlabeled comments for manual labeling and found that 45 of them were not suitable for being labeled as bullish or bearish. These results demonstrate that using a two-class sentiment analysis model to obtain more sentiments does not necessarily improve F1-score compared to using only the two-class sentiments actively labeled by users.

In Exp. 4, we collected all comments from the SPY on Stocktwits over the past two years and classified them using the FinBERT model, which provides not only positive and negative sentiments but also neutral sentiments. This addresses the issue of Exp. 3, where a two-class sentiment analysis model may classify some non-bullish and non-bearish comments as bullish or bearish. However, FinBERT is likely to classify sentiments that are not related to finance as neutral. By calculating the formula of the three-class sentiment index mentioned in the “Methodology” section, we can distinguish non-bullish or non-bearish comments on topics unrelated to finance, and reasonable sentiment indexes are obtained. The F1-score of the proposed alternative approach is higher than other methods.

In Exp. 5, we employed VADER as the sentiment analysis model, which is capable of outputting three results (positive, negative, and neutral). However, since the model is not specifically designed for the financial domain, its performance did not surpass that of Exp. 4 (FinBERT).

## Discussions

**Figure 9:**
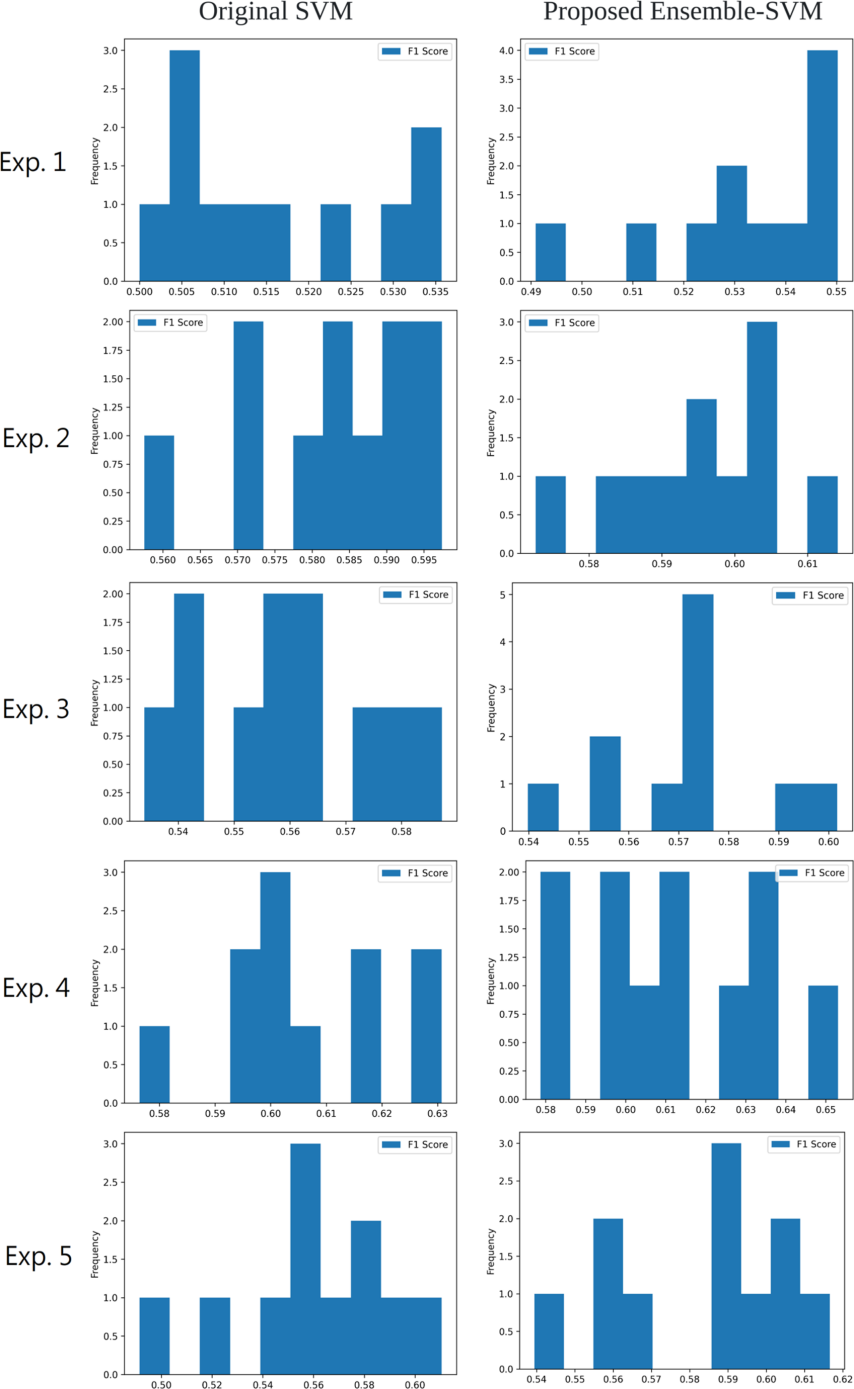
The frequency histogram of the F1-score for each experiment.

In Fig. 9, the F1-Score frequency histograms for each experiment are presented, showcasing the improvement in stock movement prediction performance achieved by the ensemble SVM implemented across all experiments. By employing the Bagging technique, the ensemble SVM in this study combines the forecasts from multiple base predictors, effectively capturing dataset diversity and enhancing the model’s generalization capabilities. This approach also reduces the likelihood of overfitting compared to using a single predictor, consequently boosting accuracy. The optimal outcome is obtained by the proposed methodology, which utilizes FinBERT for sentiment analysis and the ensemble SVM for stock price movement prediction in Exp. 4, reaching a peak F1-score of 65.30%.

In Exp. 1 and Exp. 2, we compare the F1-score improvement of stock movement prediction by adding investor sentiment. Since Gupta & Chen (2020) developed multiple two-class sentiment analyzers with accuracy between about 75–85%, and the sentiment analysis was followed by stock price movement prediction, the results showed that more comments were effective in improving the accuracy. Therefore, to compare with this method, we used a two-class sentiment analyzer with 93% accuracy in Exp. 3. However, the results of the stock price movement prediction were not satisfactory. In Gupta & Chen (2020), it is mentioned that a model with neutral sentiment may be able to reduce noise and improve accuracy. Therefore, in the method of Koukaras, Nousi & Tjortjis (2022), a model with neutral sentiment such as VADER was used for the analysis, and good results were obtained. However, since they used only 22 days in testing set, we are unable to determine its generalizability. In Exp. 5, we used our dataset—more than 10 times the size of their dataset—for prediction. The results show that this approach outperforms Exp. 3, but not necessarily better than Exp. 2 (only predicts based on user-initiated sentiment marking). In Exp. 4, we propose an alternative approach, which is to apply the FinBERT model as the sentiment analysis model. The results show that this approach achieves the best experimental results.

## Conclusions

In this article, we propose an alternative approach to sentiment analysis of Stocktwits comments for predicting stock price movements. Our proposed method utilizes the FinBERT model to reduce noise, enhance the macro-level and accuracy of sentiment indexes, and address classification errors arising from the two-class sentiment analysis model. By comparing our approach with techniques used in previous studies, we demonstrate its effectiveness. Moreover, we introduce an ensemble support vector machine model to improve the generalization ability and increase the accuracy of stock price movement predictions. We also employ a rolling window approach to prevent look-ahead bias. Overall, our study contributes to the growing body of research on sentiment-based stock price prediction and has the potential to assist investors in making informed decisions.

Despite the ideal accuracy achieved in predicting stock price movements using the proposed method, there are limitations when relying solely on sentiment. Two main directions for future improvement can be identified. First, methods that rely exclusively on social media sentiment for stock price prediction are vulnerable to intentional manipulation, such as the GameStop short squeeze in 2021. Therefore, it is necessary to establish protective mechanisms against such manipulations in the future. Second, the performance of methods that depend solely on social media sentiment for stock price prediction is limited. To enhance prediction accuracy, we plan to combine the method proposed in this article with technical analysis indicators in future research.

##  Supplemental Information

10.7717/peerj-cs.1403/supp-1 Supplemental Information 1 Sentiment analysis

10.7717/peerj-cs.1403/supp-2 Supplemental Information 2 Stock price movement prediction

10.7717/peerj-cs.1403/supp-3 Supplemental Information 3 SPY historical data

10.7717/peerj-cs.1403/supp-4 Supplemental Information 4 SPY historical data in 15 min increments

## Additional Information and Declarations

### Data Availability

The following information was supplied regarding data availability: The sentiment analysis and experiment and stock price history data are available in the Supplemental Files. The SPY messages dataset is available at Figshare: Liu, Jin-Xian (2022): SPY_stocktwits_messages.csv. figshare. Dataset. https://doi.org/10.6084/m9.figshare.20237736.v1.
